# Identification of a Novel Metastasis-Related miRNAs-Based Signature for Predicting the Prognosis of Hepatocellular Carcinoma

**DOI:** 10.1155/2021/6629633

**Published:** 2021-01-31

**Authors:** Yuan Chen, Guifu Wang, Hao Xu, Hao Wang, Dousheng Bai

**Affiliations:** ^1^Yangzhou University Medical College, Yangzhou, Jiangsu, China; ^2^Dalian Medical University, Dalian, Liaoning, China; ^3^Department of Hepatobiliary and Pancreatic Surgery, Yangzhou University Clinical Medical College, Yangzhou, Jiangsu, China

## Abstract

Hepatocellular carcinoma (HCC) is one of the most common internal malignancies worldwide and is associated with a poor prognosis. Abnormal expression of miRNAs is believed to play a role in the recurrent metastasis of HCC. However, limited studies on the role of miRNAs in HCC metastasis have been carried out. Therefore, this study is aimed at exploring the potential value of metastasis-related miRNAs (MRMs) in HCC. We retrieved MRMs were from the Human Cancer Metastasis Database. Differential miRNAs were identified for tumor samples of HCC patients and normal samples based on the TCGA database. Further, univariate and multivariate Cox regression analyses were used to screen MRMs known to be independent prognostic factors in HCC. These MRMs were then used to build a prognostic signature. All patients were classified into high-risk and low-risk groups based on the median of the signature scores. Moreover, GO and KEGG pathway enrichment analyses were performed to predict the function of these MRMs. Finally, a nomogram was constructed to predict the OS of patients at 1, 2, and 3 years. In our study, a total of seven prognostic MRMs (miR-140-3p, miR-9-5p, miR-942-5p, miR-324-3p, miR-29c-5p, miR-551a, and miR-149-5p) were identified and used for constructing the prognostic signature based on the training cohort. Patients in the low-risk HCC group showed better overall survival (OS) than those in the high-risk group. The results were validated using the validation cohort. In summary, the findings of this study provide evidence that MRMs-based prognostic signature is an independent biomarker in the prognosis of HCC patients.

## 1. Introduction

Hepatocellular carcinoma (HCC) is the fifth most common malignancy in the world and the second leading cause of cancer-related deaths [1]. Despite significant advances in surgery, liver transplantation, and interventional therapy, the mortality rate remains high as a result of the poor prognosis of HCC patients due to late diagnosis [2]. Tumor metastasis plays a major role in the poor prognosis of HCC patients. Although HCC has many staging systems, such as the American Joint Committee on Cancer (AJCC) TNM stage and the Barcelona-Clinic Liver Cancer (BCLC) system, approaches for evaluation of survival and prognosis of patients are limited [3]. Therefore, there is a need to develop a novel metastasis-related signature for improved prediction of prognosis of HCC patients.

MiRNAs are a class of small endogenous single-stranded noncoding RNAs. MiRNAs downregulate gene expression through targeted degradation of mRNAs or by inhibiting the translation process [4]. Recently, studies have reported overexpressed miRNA-96-5p promotes gastric cancer cell proliferation [5], miR-26 affects apoptosis of hepatocellular carcinoma [6], miR-23a promotes invasion of glioblastoma [7], and miR-17-5p promotes angiogenesis in nasopharyngeal carcinoma [8]. Moreover, previous studies have found that miRNAs are downregulated or overexpressed in several cancer types, including breast cancer [9], colorectal cancer [10], and HCC [11]. It has been reported that miRNA dysregulation plays a crucial role in the metastasis of tumors [12–14]. However, the role of metastasis-related miRNA (MRMs) in HCC and its prognosis role has not been explored fully.

In this work, we examined the correlation between the expression of MRMs and the clinical data of HCC patients. A seven-MRM prognostic signature based on the training cohort was constructed for the prediction of the overall survival (OS) for HCC patients. Moreover, we constructed a nomogram to predict the OS of patients at 1, 2, and 3 years. The study provides a potential prognostic indicator for patients with HCC who have undergone surgical resection based on The Cancer Genome Atlas (TCGA) database.

## 2. Material and Methods

### 2.1. Data Acquisition

A total of 710 MRMs were retrieved from the Human Cancer Metastasis Database (https://hcmdb.i-sanger.com). The MRMs were from recurrent cases after undergoing living donor liver transplantation (LDLT). Similarly, we retrieved miRNA expression and clinical information of 375 HCC patients from the TCGA database (https://cancergenome.nih.gov). A total of 344 cases were enrolled for the study after deleting samples with no survival data or with a survival time less than 30 days.

### 2.2. Differentially Expressed miRNAs (DEMs)

The limma package in R software was used for the analysis of differential expression of MRMs in HCC tissues and adjacent nontumor tissues. A cut-off value of ∣logFC∣ >0.5 and FDR <0.05 was used. Pheatmap and ggpubr packages in R software were used to generate volcano plots, heatmaps, and box plots.

### 2.3. Construction of Prognostic Signature

The screened clinical samples were randomly grouped into training cohort (*n* = 172) and validation cohort (*n* = 172). The clinicopathological features of the training cohort and validation cohort were recorded (Table 1). Univariate Cox regression analysis was used to identify MRMs in the training cohort which were highly correlated with HCC OS. Further, multivariate Cox regression analysis was used to filter out independent prognostic factors from this group. A survival plot of the screened miRNAs was drawn, and a prognostic signature was established using the metastasis-associated prediction formula. The risk score of each patient was calculated based on the prognostic signature constructed from the training cohort. Patients in the training cohort were divided into a high-risk group and low-risk group based on the median risk score. The Kaplan–Meier (K–M) methods were used for survival analysis of the high-risk and low-risk groups. Subsequently, the role of the prediction signature was evaluated by calculating the area under the curve (AUC) using the receiver operator characteristic (ROC) curve.

### 2.4. Validation of the Prognostic Signature

Patients in the validation cohort were grouped into high-risk and low-risk groups based on the median risk score of the training cohort. The risk score and formula of the validation cohort were evaluated. Further, we evaluated the OS of the high- and low-risk groups. In addition, the prognostic value of the predictive signature was evaluated using the ROC curve.

### 2.5. GO and KEGG Pathway Enrichment Analysis

To investigate significantly enriched functions and important pathways in which MRMs play a role, we performed Gene Ontology (GO) and Kyoto Encyclopedia of Genes and Genomes (KEGG) pathway enrichment analysis and the results were visualized via R software. Corrected *P* value <0.05 was considered statistically significant.

### 2.6. PPI Network Analysis

Protein-protein interaction (PPI) network analysis data analyzed from STRING online website (https://string-db.org/). Further analysis and visualization of protein interaction network data were completed by Cytoscape software (version 3.7.2).

### 2.7. Nomogram Model Construction

We combined the risk score and the corresponding clinical variables (age, TNM stage, grade) to construct a nomogram for the prediction of the OS of patients at 1, 2, and 3 years. The nomogram was used for effective use and visualization of the prognostic signature.

### 2.8. Statistical Analysis

All gene expression data were normalized by log2 transformation. We used R software (version 3.6.2) to perform all statistical analyses. In all analyses, *P* value <0.05 was considered statistically significant.

## 3. Result

### 3.1. Differentially Expressed MRMs

**Figure 1:**
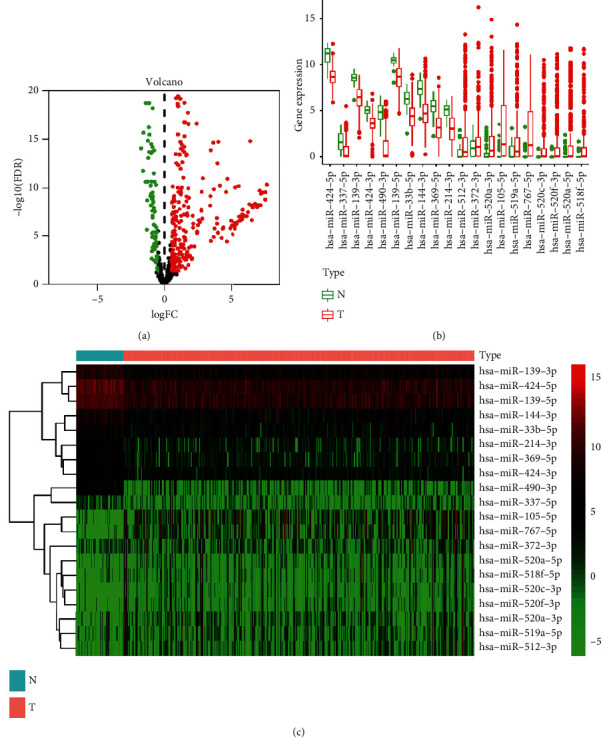
Differentially expressed MRMs between HCC and normal tissue. (a) Volcano plots showing miRNAs expression. (b) and (c) Top 10 upregulated and top 10 downregulated miRNAs.

Among the 710 metastasis-associated genes, 335 differentially expressed MRMs were identified in the analysis of HCC and normal tissue. Volcano plots were used to visualize expression patterns of differentially expressed MRMs between HCC and nontumor tissues (Figure 1(a)). Further, a heat map and box plot were constructed using the top 10 upregulated miRNA and the top 10 downregulated miRNA (Figures 1(b) and 1(c)).

### 3.2. Construction of Prognostic Signature

**Figure 2:**
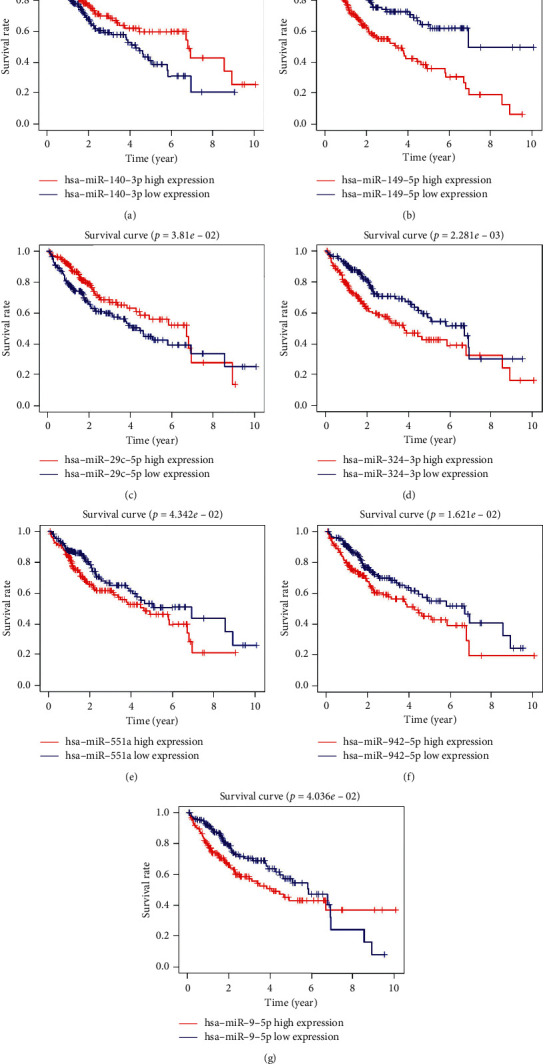
Survival analysis for each prognostic MRM by the Kaplan–Meier plots. (a) miR-140-3p, (b) miR-149-5p, (c) miR-29c-5p, (d) miR-324-3p, (e) miR-551a, (f) miR-942-5p, and (g) miR-9-5p.

Only 26 of the 335 MRMs were linked to the prognosis of HCC patients using univariate Cox regression analysis. Further, we performed multivariate analysis on the 26 MRMs. As a result, miR-140-3p, miR-9-5p, miR-942-5p, miR-324-3p, miR-29c-5p, miR-551a, and miR-149-5p were picked out as potential independent prognostic predictors in HCC. In addition, we validated the prognostic significance of the genes using a survival curve. High expression levels of miR-9-5p, miR-942-5p, miR-324-3p, miR-551a, and miR-149-5p were significantly correlated with poor OS. On the other hand, upregulation of miR-140-3p and miR-29c-5p indicated better survival of HCC patients (Figures 2(a)–2(g)).

**Figure 3:**
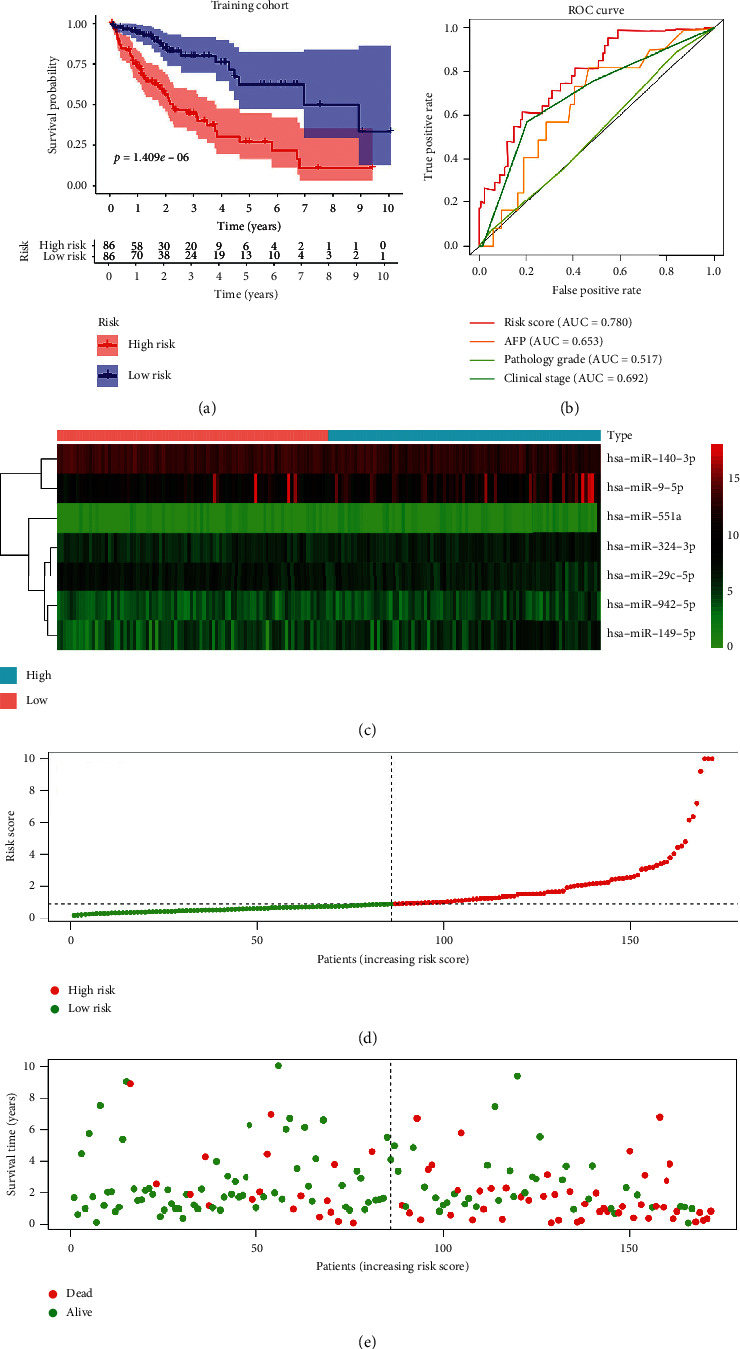
Risk score of HCC patients in the TCGA training cohort. (a) The Kaplan–Meier survival analysis of HCC patients. (b) ROC curve for the risk score and clinicopathological characteristics of HCC patients. (c) Heatmap of the expression pattern of the seven-MRM signature between high-risk and low-risk groups. (d) Risk score distribution in each HCC patient. (e) The OS and OS status for the high-risk and low-risk groups.

Multivariate Cox regression analysis results are shown in Table 2. The prognostic signature was constructed using Cox regression analysis results. The risk score for each sample was calculated using the following formula: risk score = (0.36809^*∗*^ the expression of miR-942-5p) + (0.27523^*∗*^ the expression of miR-324-3p) + (0.26632^*∗*^ the expression of miR-551a) + (0.26487^*∗*^ the expression of miR-29c-5p) + (0.10317^*∗*^ the expression of miR-9-5p)−(0.26632^*∗*^ the expression of miR-29c-5p)−(0.66679^*∗*^ the expression of miR-140-3p). Further, we grouped the training cohort into a high-risk group (*n* = 86) and low-risk group (*n* = 86) using the median value of the risk score. The two groups were analyzed to identify the key role of the prognostic signature in predicting the prognosis of HCC. Survival analysis showed significantly low survival of samples with a high-risk score compared with samples with a low-risk score (Figure 3(a)). As shown in (Figure 3(b)), the ROC curve showed that the risk score had a significant prognostic value in HCC patients (AUC = 0.780). And the area under the curve was larger than other clinicopathological characteristics, such as AFP, pathology grade, and clinical stage. The heatmap showed the expression pattern of these seven prognostic miRNAs (Figure 3(c)), and the samples were ranked in ascending order of the parameters (Figure 3(d)). The risk score ordered the scatterplot of patient survival status (Figure 3(e)).

### 3.3. Validation of the Prognostic Signature

**Figure 4:**
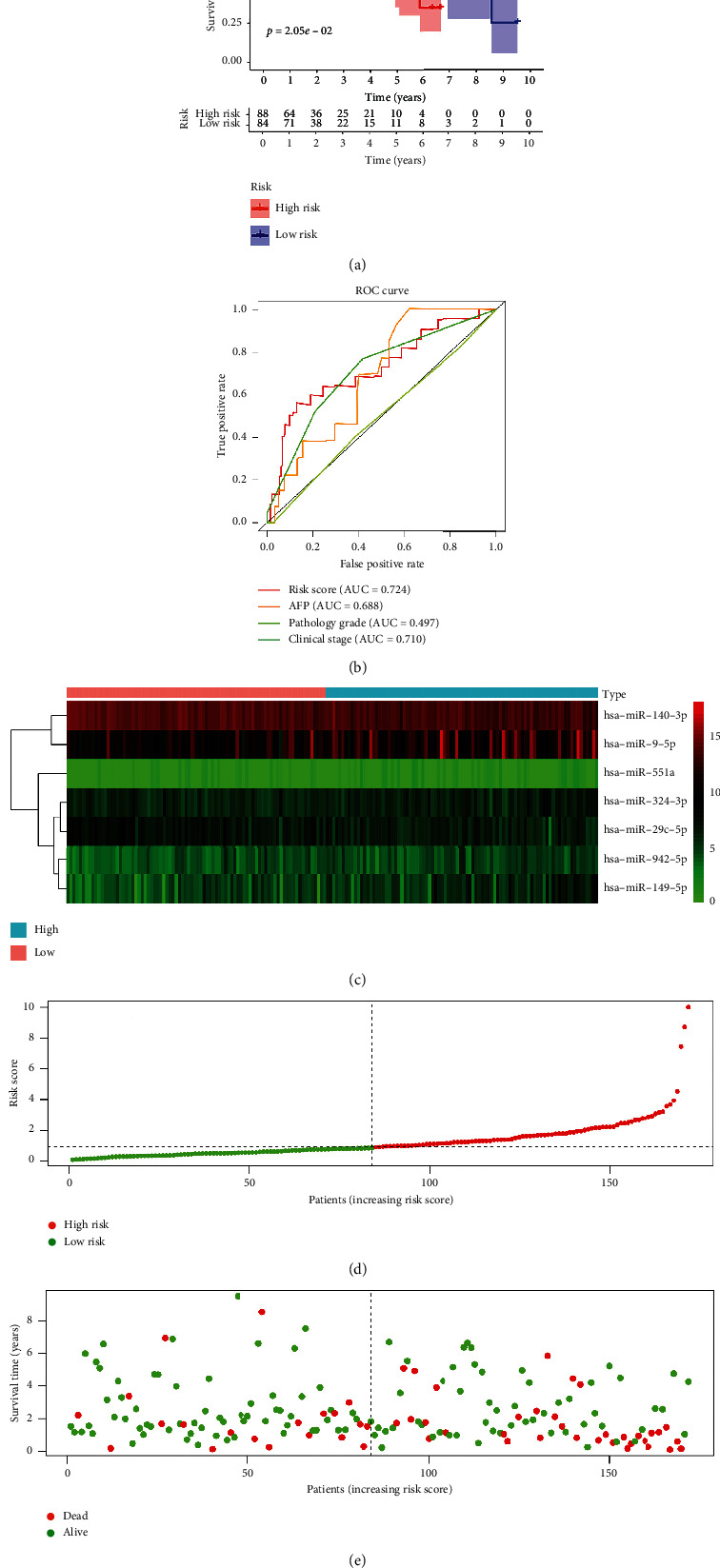
Risk score of HCC patients in the TCGA test cohort. (a) The Kaplan–Meier survival analysis of HCC patients. (b) ROC curve for the risk score and clinicopathological characteristics of HCC patients. (c) Heatmap of the expression pattern of the seven-MRM signature for the high-risk and low-risk groups. (d) Risk score distribution in each HCC patient. (e) The OS and OS status for the high-risk and low-risk groups.

Further, the cut-off value used for the training cohort was used to group the validation cohort into a high-risk group (*n* = 86) and a low-risk group (*n* = 84). Results obtained from the analysis of the validation cohort were similar to the training cohort results. The prognosis of the sample with a high-risk score significantly poor compared to the samples with low-risk score **(**Figure 4(a)**)**. In addition, the ROC curve (Figure 4(b)) showed that the risk score was effective for the prognosis of HCC patients (AUC = 0.724). Similarly, the area under the curve was larger than other clinicopathological characteristics, such as AFP, pathology grade, and clinical stage. The risk score for the validation cohort was calculated as described in the training cohort. The distribution of prognostic miRNAs between both groups was presented as a heatmap (Figure 4(c)). The distribution of risk score, the OS, and OS status were as shown (Figures 4(d) and 4(e)).

### 3.4. GO and KEGG Pathway Enrichment Analysis

**Figure 5:**
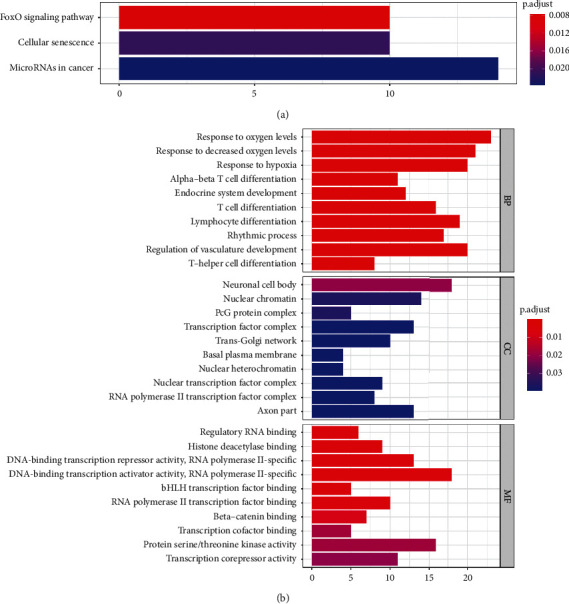
GO and KEGG pathway enrichment analysis. (a) Bar plot for signaling pathways in which MRMs play a role in KRGG. (b) GO analysis on the association of BP, CC, and MF with MRMs (BP: biological process, CC: cell component, and MF: molecular function).

We used three databases (TargetScan (http://www.targetscan.org/) [15], miRDB (http://www.mirdb.org/) [16], miRTarBase (http://mirtarbase.mbc.nctu.edu.tw/) [17] to identify potential target genes of prognostic miRNAs. We selected the common target genes in these three databases. However, the target genes of miR-551a and miR-29c-5p were selected based on two datasets because there are too few common target genes in the other dataset. A total of 260 target genes were used for subsequent analysis. We performed GO enrichment and KEGG pathway enrichment analyses to identify biological functions and pathways where the identified genes are implicated. GO and KEGG enrichment analysis results were as shown in Figures 5(a) and 5(b). KEGG pathway analysis showed that target genes regulated several biological pathways, including FoxO signaling pathway, cellular senescence, and miRNAs in cancer. GO enrichment analysis showed that target genes are mainly involved in response to oxygen levels, decreased oxygen level, and hypoxia at the biological process level. Neuronal cell body, nuclear chromatin, and PcG protein complex were the most enriched cellular components. Molecular function, DNA-binding transcription activator/repressor activity and RNA polymerase II-specific, and histone deacetylase binding were significantly enriched in HCC samples compared with normal cells.

### 3.5. PPI Network Analysis

**Figure 6:**
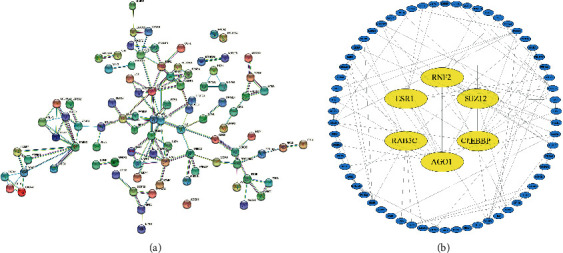
PPI network analysis. (a) PPI networks among the target genes of 7 prognostic miRNAs. (b) Core target genes of the protein interaction network.

We used the STRING online website to analyze the potential interactions between the 260 target genes of the 7 prognostic miRNAs, and the minimum required interaction score was set at 0.9 (Figure 6(a)). Also, the above results were further analyzed and visualized by Cytoscape software. The top 6 nodes with greater degrees were displayed in the middle of the image, including RNF2, ESTR1, RAB5C, AGO1, CREBBP, and SUZ12 (Figure 6(b)).

### 3.6. Nomogram Model Construction

**Figure 7:**
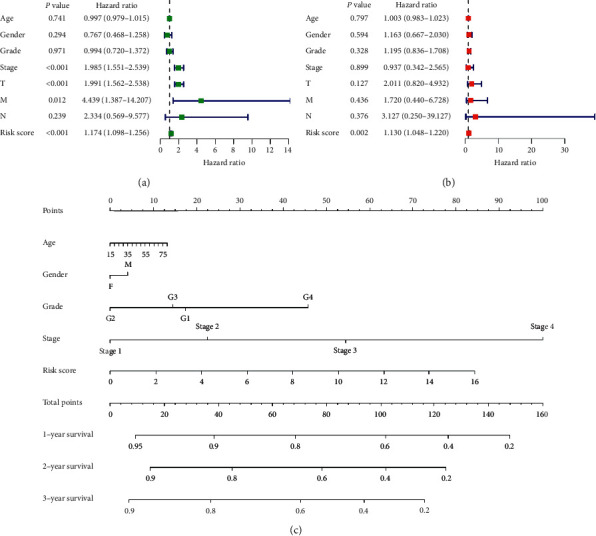
Validation of the prognostic signature. (a) Univariate Cox regression analysis of risk score (RS) in HCC training cohort. (b) Multivariate Cox regression analysis of risk score in HCC training cohort. (c) Nomogram prediction model established using risk signature and clinical and pathological parameters.

Moreover, we verified the effectiveness of the prognostic signature in predicting the prognosis of HCC patients using univariate Cox analysis and multivariate Cox analysis for the training cohort (Figures 7(a) and 7(b)**)**. The results showed that the prognostic signature was an independent prognostic factor for HCC. Further, we constructed a nomogram to establish a more sensitive prediction signature to predict the prognosis of HCC **(**Figure 7(c)). The prognostic signature, age, sex, tumor grade, and TNM stage were incorporated into the nomogram model. The total score of the nomogram can be used for the prediction of the 1-, 2-, and 3-year OS of patients with HCC.

### 3.7. The Relationship between MRMs and Clinical Variables

**Figure 8:**
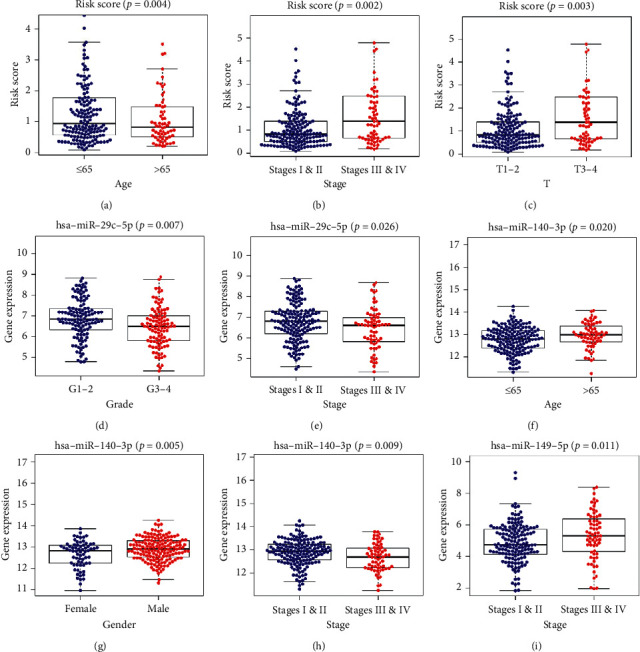
The clinicopathological significance of risk score and MRMs in HCC. (a) Risk score and age. (b) Risk score and pathology grade. (c) Risk score and clinical stage. (d) miR-29c-5p expression level and pathology grade. (e) miR-29c-5p expression level and clinical stage. (f) miR-140-3p expression level and age. (g) miR-140-3p expression level and gender. (h) miR-140-3p expression level and clinical stage. (i) miR-149-5p expression level and clinical stage.

Analysis of the correlation between clinical variables and seven MRMs showed that the risk score of elderly patients (age >65 years old) was lower compared with that of young patients (age ≤65 years old). Furthermore, the risk score of patients with advanced tumors (stages III ∼IV or T3∼T4) was higher than that of patients with early-stage tumors (Figures 8(a)–8(c)). miR-29c-5p was significantly correlated to T stage, grade, and stage; miR-140-3p was significantly correlated to T stage, age, gender, and stage whereas miR-149-5p was significantly correlated to T stage and age (Figures 8(d)–8(i)).

## 4. Discussion

HCC is one of the most lethal cancers worldwide [18]. Hepatitis-cirrhosis-HCC is the main cause of most liver cancer cases. Most patients with HCC have a history of hepatitis B or C infection [19]. Surgical resection is still the mainstream treatment approach for HCC; however, the postoperative survival of HCC patients is low due to the high incidence of metastasis [20]. Previous studies report that miRNA dysregulation is associated with metastasis of liver cancer [21–23]. However, the prognostic value of metastasis-related miRNAs in HCC has not been fully elucidated. Therefore, the identification of miRNAs and the targets associated with HCC metastasis may provide promising therapeutic avenues.

In this study, we retrieved miRNA expression profiles and clinical information of 373 HCC samples and 50 normal samples from the TCGA database. Further, we selected and analyzed differentially expressed MRMs in HCC. We identified seven MRMs (miR-140-3p, miR-9-5p, miR-942-5p, miR-324-3p, miR-29c-5p, miR-551a, and miR-149-5p) as potential independent prognostic predictors for HCC through univariate and multivariate analyses. Further, a prediction signature based on the 7 MRMs showed good performance in predicting the OS of HCC. GO and KEGG enrichment analysis indicated that the seven MRMs may be involved in metastasis of HCC through the regulation of different pathways. We constructed a more sensitive prediction signature using the nomogram method to predict the prognosis of HCC.

Our results suggest that the screened MRMs are independent prognostic predictors. Out of the differentially expressed miRNAs, miR-140-3p, miR-9-5p, miR-324-3p, and miR-149-5p were implicated in HCC metastasis. On the other hand, miR-942-5p, miR-29c-5p, and miR-551 were reported in other cancer types but not in HCC. For instance, high expression levels of miR-140-3p inhibit EMT, invasion, and metastasis of HCC by targeting GRN [24], whereas miR-9-5p and miR-149-5p promote HCC progression [25, 26]. On the contrary, upregulation of miR-551a by dimethoxy curcumin hinders metastasis of ovarian cancer cells [27]. miR-135b-5p modulates APC gene in both diffuse and intestinal gastric cancer subtypes [28]. GO enrichment analysis of seven-MRMs were associated with changes in oxygen levels in biological processes. Notably, hypoxia is associated with metastasis of HCC [29, 30]. The FoxO signaling pathway was identified as the significantly enriched pathway through KEGG analysis. FoxO is reported to play a vital role in the metastasis of HCC [31]. PPI analysis revealed those seven miRNAs' target genes with the strongest protein interactions. Moreover, it has been reported that the abnormal expression of RNF2, AGO1, CREBBP, and SUZ12 is closely related to the invasion and metastasis of HCC [32–35]. These results indicate that MRMs are associated with metastasis of HCC. However, this study has a few shortcomings. We only validated the seven miRNAs prognostic signature in the validation cohort; therefore, large independent studies are needed to verify the effectiveness of this signature. In addition, we selected MRMs related to the overall survival of HCC patients and did not do further experiments to explore their mechanism in metastasis.

In conclusion, our study evaluated expression profiles of metastasis-related miRNAs retrieved from the TCGA database and established a prediction signature. The seven-miRNA molecular signature accurately predicts the prognosis of HCC; therefore, it has important implications in clinical practice.

## Figures and Tables

**Table 1:** Clinicopathological characteristics of training cohort and validation cohort.

	Overall	Training cohort	Validation cohort	*p*
*n*	344	172	172	
Age (mean (SD))	59.35 (13.19)	60.21 (13.30)	58.50 (13.07)	0.23
Gender = male (%)	236 (68.6)	118 (68.6)	118 (68.6)	1

Pathology grade (%)				0.406
G1	53 (15.4)	25 (14.5)	28 (16.3)	
G2	160 (46.5)	83 (48.3)	77 (44.8)	
G3	114 (33.1)	52 (30.2)	62 (36.0)	
G4	13 (3.8)	9 (5.2)	4 (2.3)	
Unknown	4 (1.2)	3 (1.7)	1 (0.6)	

Clinical stage (%)				0.575
Stage I	162 (47.1)	74 (43.0)	88 (51.2)	
Stage II	77 (22.4)	42 (24.4)	35 (20.3)	
Stage III	3 (0.9)	2 (1.2)	1 (0.6)	
Stage IIIA	59 (17.2)	30 (17.4)	29 (16.9)	
Stage IIIB	9 (2.6)	4 (2.3)	5 (2.9)	
Stage IIIC	9 (2.6)	4 (2.3)	5 (2.9)	
Stage IV	1 (0.3)	0 (0.0)	1 (0.6)	
Stage IVB	2 (0.6)	2 (1.2)	0 (0.0)	
Unknown	22 (6.4)	14 (8.1)	8 (4.7)	

T classification (%)				0.711
T1	169 (49.1)	80 (46.5)	89 (51.7)	
T2	82 (23.8)	45 (26.2)	37 (21.5)	
T2a	1 (0.3)	0 (0.0)	1 (0.6)	
T2b	1 (0.3)	0 (0.0)	1 (0.6)	
T3	42 (12.2)	23 (13.4)	19 (11.0)	
T3a	26 (7.6)	12 (7.0)	14 (8.1)	
T3b	7 (2.0)	2 (1.2)	5 (2.9)	
T4	13 (3.8)	8 (4.7)	5 (2.9)	
TX	1 (0.3)	1 (0.6)	0 (0.0)	
Unknown	2 (0.6)	1 (0.6)	1 (0.6)	

M classification (%)				0.455
M0	248 (72.1)	119 (69.2)	129 (75.0)	
M1	3 (0.9)	2 (1.2)	1 (0.6)	
MX	93 (27.0)	51 (29.7)	42 (24.4)	

N classification (%)				0.407
N0	241 (70.1)	115 (66.9)	126 (73.3)	
N1	3 (0.9)	1 (0.6)	2 (1.2)	
NX	99 (28.8)	55 (32.0)	44 (25.6)	
Unknown	1 (0.3)	1 (0.6)	0 (0.0)	

**Table 2:** Results of multivariate Cox regression analysis for MRMS in the training cohort.

MRMs	Coefficient	HR	HR. 95L	HR. 95H	*P* value
hsa-miR-942-5p	0.36809	1.44497	1.09978	1.89851	0.00822
hsa-miR-324-3p	0.27523	1.31683	0.92256	1.87961	0.12953
hsa-miR-551a	0.26632	1.30516	1.04248	1.63402	0.02019
hsa-miR-149-5p	0.20294	1.22500	1.02264	1.46739	0.02760
hsa-miR-9-5p	0.10317	1.10868	0.99782	1.23186	0.05494
hsa-miR-29c-5p	−0.26487	0.76730	0.56868	1.03531	0.08311
hsa-miR-140-3p	−0.66679	0.51335	0.32004	0.82342	0.00568

## Data Availability

The data are available at the Human Cancer Metastasis Database (https://hcmdb.i-sanger.com), TCGA database (https://cancergenome.nih.gov), TargetScan (http://www.targetscan.org/), miRDB (http://www.mirdb.org/), and miRTarBase (http://mirtarbase.mbc.nctu.edu.tw/).

## References

[1] Chang R.-M., Xiao S., Lei X., Yang H., Fang F., Yang L.-Y. (2017). miRNA-487a promotes proliferation and metastasis in hepatocellular carcinoma. *Clinical Cancer Research*.

[2] Sapisochin G., Bruix J. (2017). Liver transplantation for hepatocellular carcinoma: outcomes and novel surgical approaches. *Nature Reviews Gastroenterology & Hepatology*.

[3] Ma X., Gu J., Wang K. (2019). Identification of a molecular subtyping system associated with the prognosis of Asian hepatocellular carcinoma patients receiving liver resection. *Scientific Reports*.

[4] Kuersten S., Goodwin E. B. (2003). The power of the 3′ UTR: translational control and development. *Nature Reviews Genetics*.

[5] He X., Zou K. (2020). MiRNA-96-5p contributed to the proliferation of gastric cancer cells by targeting FOXO3. *The Journal of Biochemistry*.

[6] Jin F. F., Wang Y. B., Li M. Z. (2017). MiR-26 enhances chemosensitivity and promotes apoptosis of hepatocellular carcinoma cells through inhibiting autophagy. *Cell Death & Disease*.

[7] Yachi K., Tsuda M., Kohsaka S. (2018). miR-23a promotes invasion of glioblastoma via HOXD10-regulated glial-mesenchymal transition. *Signal Transduction and Targeted Therapy*.

[8] Duan B., Shi S., Yue H. (2019). Exosomal miR-17-5p promotes angiogenesis in nasopharyngeal carcinoma via targeting BAMBI. *Journal of Cancer*.

[9] McGuire A., Brown J. A. L., Kerin M. J. (2015). Metastatic breast cancer: the potential of miRNA for diagnosis and treatment monitoring. *Cancer and Metastasis Reviews*.

[10] Dong J., Tai J. W., Lu L.-F. (2019). miRNA-microbiota interaction in gut homeostasis and colorectal cancer. *Trends in Cancer*.

[11] Shyu Y. C., Lee T. L., Lu M. J. (2016). miR-122-mediated translational repression of PEG10 and its suppression in human hepatocellular carcinoma. *Journal of Translational Medicine*.

[12] Baranwal S., Alahari S. K. (2010). miRNA control of tumor cell invasion and metastasis. *International Journal of Cancer*.

[13] Torres S., Garcia-Palmero I., Bartolomé R. A. (2017). Combined miRNA profiling and proteomics demonstrates that different miRNAs target a common set of proteins to promote colorectal cancer metastasis. *The Journal of Pathology*.

[14] Wang T., Xu H., Qi M., Yan S., Tian X. (2018). miRNA dysregulation and the risk of metastasis and invasion in papillary thyroid cancer: a systematic review and meta-analysis. *Oncotarget*.

[15] Lewis B. P., Burge C. B., Bartel D. P. (2005). Conserved seed pairing, often flanked by adenosines, indicates that thousands of human genes are microRNA targets. *Cell*.

[16] Wong N., Wang X. (2015). miRDB: an online resource for microRNA target prediction and functional annotations. *Nucleic Acids Research*.

[17] Hsu S.-D., Tseng Y.-T., Shrestha S. (2014). miRTarBase update 2014: an information resource for experimentally validated miRNA-target interactions. *Nucleic Acids Research*.

[18] Budny A., Kozlowski P., Kaminska M. (2017). [Epidemiology and risk factors of hepatocellular carcinoma]. *Polski Merkuriusz Lekarski*.

[19] Ghouri Y. A., Mian I., Rowe J. H. (2017). Review of hepatocellular carcinoma: epidemiology, etiology, and carcinogenesis. *Journal of Carcinogenesis*.

[20] Orcutt S. T., Anaya D. A. (2018). Liver resection and surgical strategies for management of primary liver cancer. *Cancer Control*.

[21] Zhou H.-C., Fang J.-H., Shang L.-R. (2016). MicroRNAs miR-125b and miR-100 suppress metastasis of hepatocellular carcinoma by disrupting the formation of vessels that encapsulate tumour clusters. *The Journal of Pathology*.

[22] Liu Z., Li W., Pang Y. (2018). SF3B4 is regulated by microRNA-133b and promotes cell proliferation and metastasis in hepatocellular carcinoma. *Ebiomedicine*.

[23] Chen Y., Guo Y., Li Y. (2019). miR300 regulates tumor proliferation and metastasis by targeting lymphoid enhancerbinding factor 1 in hepatocellular carcinoma. *International Journal of Oncology*.

[24] Zhang Q. Y., Men C. J., Ding X. W. (2019). Upregulation of microRNA‐140‐3p inhibits epithelial‐mesenchymal transition, invasion, and metastasis of hepatocellular carcinoma through inactivation of the MAPK signaling pathway by targeting GRN. *Journal of Cellular Biochemistry*.

[25] Dong X., Wang F., Xue Y. (2019). MicroRNA95p downregulates Klf4 and influences the progression of hepatocellular carcinoma via the AKT signaling pathway. *International Journal of Molecular Medicine*.

[26] Liu G., Yin L., Ouyang X., Zeng K., Xiao Y., Li Y. (2020). M2 macrophages promote HCC cells invasion and migration via miR-149-5p/MMP9 signaling. *Journal of Cancer*.

[27] Du Z., Sha X. (2017). Demethoxycurcumin inhibited human epithelia ovarian cancer cells’ growth via up-regulating miR-551a. *Tumour Biology*.

[28] Magalhaes L., Quintana L. G., Lopes D. C. F. (2018). APC gene is modulated by hsa-miR-135b-5p in both diffuse and intestinal gastric cancer subtypes. *BMC Cancer*.

[29] Legendre C., Hori T., Loyer P. (2009). Drug-metabolising enzymes are down-regulated by hypoxia in differentiated human hepatoma HepaRG cells: HIF-1*α* involvement in CYP3A4 repression. *European Journal of Cancer*.

[30] Liu Z., Wang Y., Dou C. (2018). Hypoxia-induced up-regulation of VASP promotes invasiveness and metastasis of hepatocellular carcinoma. *Theranostics*.

[31] Hou Y. Q., Yao Y., Bao Y. L. (2016). Juglanthraquinone C induces intracellular ROS increase and apoptosis by activating the Akt/Foxo signal pathway in HCC cells. *Oxidative Medicine and Cellular Longevity*.

[32] Qu C., Qu Y. (2017). Down-regulation of salt-inducible kinase 1 (SIK1) is mediated by RNF2 in hepatocarcinogenesis. *Oncotarget*.

[33] Wang M., Zhang L., Liu Z. (2018). AGO1 may influence the prognosis of hepatocellular carcinoma through TGF-*β* pathway. *Cell Death & Disease*.

[34] Wen D. Y., Lin P., Liang H. W. (2018). Up-regulation of CTD-2547G23.4 in hepatocellular carcinoma tissues and its prospective molecular regulatory mechanism: a novel qRT-PCR and bioinformatics analysis study. *Cancer Cell International*.

[35] Xue C., Wang K., Jiang X. (2019). The down-regulation of SUZ12 accelerates the migration and invasion of liver cancer cells via activating ERK1/2 pathway. *Journal of Cancer*.

